# Self-Plagiarism and Redundant Publications: A True Scientific Misconduct

**DOI:** 10.3390/tomography11090102

**Published:** 2025-09-02

**Authors:** Emilio Quaia

**Affiliations:** Department of Radiology, University of Padova, Via Giustiniani 2, 35128 Padova, Italy; emilio.quaia@unipd.it; Tel.: +39-049-8212375

This editorial provides insights on plagiarism, self-plagiarism, and redundant publications, which all represent a serious and common form of misconduct in research. The Committee on Publication Ethics (COPE) recommends extremely rigid management of redundant publication and provides some general guidelines on how to manage this issue. Pressure to publish represents the most common reason why authors produce redundant publications despite leading to serious consequences to authors and potentially hampering their academic career. Education on ethical conduct in scientific research and author knowledge of the possible consequences represent the most effective solutions to this type of scientific misconduct.

Plagiarism is the unacknowledged copying or attempt to misattribute original authorship, whether of ideas, texts, or results, usually manifesting in large parts of the text having been cut-and-pasted without appropriate attribution. Among research misconducts, plagiarism also encompasses data fabrication and falsification, as defined by the Office of Research Integrity of the US Department of Health and Human Services [1,2], and it is the most common form of misconduct [2]. These actions are considered serious breaches of research ethics, bringing significant consequences for researchers, institutions, and the credibility of the whole scientific community.

Self-plagiarism and redundant publications, while related, are distinct ethical issues in academic and scientific writing. Self-plagiarism, also defined as text-recycling or text overlap, involves reusing one’s own previously published work, either verbatim or with minor modifications, without proper attribution. While not considered theft in the same way plagiarism of another’s work is, self-plagiarism is still a form of academic misconduct and may have serious consequences.

Redundant publication, also known as duplicate publication, occurs when an author reuses substantial parts of their own published work without providing the appropriate references. This can range from publishing an identical paper in multiple journals, to only adding a small amount of new data to a previously published paper. The extreme expression of redundant publication is the so-called “salami slices”, also known as salami publishing, which corresponds to the unethical practice of dividing a single, large research study into multiple smaller publications to inflate the number of published articles, potentially misleading the scientific community and distorting the research record. The manuscript section that is most often involved in redundant publication allegations corresponds to the materials and methods section, since this section may be used for different studies simply by changing the patient population. Both self-plagiarism and redundant publications are generally considered unethical as they distort the academic record, waste resources, and can mislead readers. The Committee on Publication Ethics (COPE) recommends extremely rigid management of redundant publication, including informing the author’s superior or the person responsible for research governance and determining if appropriate actions, such as a retraction or a statement of duplicate publication, are necessary [3,4]. COPE does not recommend using a specific percentage of duplication or overlap as a benchmark for conducting an investigation. Instead, COPE suggests that the editor should look at the nature and degree of duplication [5]. Moreover, according to COPE, duplication spread across the paper in short phrases may be less of a concern than duplication concentrated in several paragraphs or sections. Similarly, the limited use of identical, or nearly identical, phrases describing common methodology or previous research may not require further investigation [5]. In this regard, there are several examples in which text redundancy can be considered acceptable, for example, papers in some fields that require reporting that is formulaic in structure or that is likely to produce plots and figures that are similar in format, or a student who duplicates his work from his own thesis or dissertation, depending on the journal’s policies on what is considered a prior formal publication (for example, a thesis deposited in an institutional repository, which is often a required practice by the institution) [5]. Moreover, re-analysis of research data is also a normally accepted approach, such as when new analysis tools or new research questions have emerged after the original publication. Some research data collection protocols are also, from the very beginning, designed to generate several independent studies. For example, when an additional series of corresponding measurements would be very difficult to carry out or even be less ethical than using the original dataset several times (e.g., the repeated injection of a contrast agent or injection of radioactive substances in healthy volunteers). These are only a few examples of potentially acceptable behaviors which should not be used to conceal other types of behaviors that fully match redundant publication.

Redundant publication even has a significant influence on the growing phenomenon of scientific paper retractions. Scientific paper retraction is a relevant issue in scientific literature and is generally due to errors, misconduct, or ethical violations [6]. Retractions are crucial for maintaining the integrity of the scientific record and preventing the spread of flawed or misleading information. Among misconduct behaviors, plagiarism is one of the most relevant reasons for paper retractions and, besides ethical violations, redundant publications represent a further relevant reason for paper retractions.

Plagiarism or self-plagiarism detection software tools, including Grammarly, Copyleaks, Quetext, Scribbr, and Turnitin, are extensively employed not only by scientific journals but even in high schools and universities to detect student misconduct. These tools check for similarities between the current work and previously published content, helping to identify instances where plagiarism or self-plagiarism might occur. Generally, a similarity score of 15–20% or higher might warrant closer examination, especially in academic contexts. However, the acceptable percentage can vary significantly depending on the specific context, the nature of the work, and the guidelines of the institution or publication. Some journals may have stricter thresholds, such as a threshold of 10%.

To avoid redundant (duplicate) publication, researchers should ensure each manuscript addresses a unique research question, clearly cites previous work, and informs journals about related publications. Transparency with editors and proper citation practices are absolutely essential and represent the basics of scientific writing. Unfortunately, pressure to publish is often overwhelming and authors may be unable to produce completely new studies in a short period of time, and often use previous data to generate further papers to address universities and their chief expectancy, pressure for career progression, or funding acquisition.

An allegation of redundant publication may exclude all the authors of a manuscript from future submissions to a journal. Moreover, the author’s Department Chair or Dean may be informed, an internal investigation may be launched, the local newspaper might report on scientific misconduct by faculty members, and the repercussions may affect a person’s promotion, tenure, and reputation [7]. The instructions to authors should state the journal’s policy on redundant publications [3]. Signing copyright forms is part of the publication process and authors may sign them without much thought about future consequences [7]. Authors should be aware that published text, notes, and figures have a copyright associated contract which must be respected.

Although plagiarism, self-plagiarism, and redundant publications are well known by authors, they are frequently detected before the manuscript revision cycle. Besides the educational aspects of raising awareness on publication ethics and responsible research conduct of researchers and journal publishers—which are always essential, in my own opinion—the only way to avoid this type of misconduct is the knowledge of the possible consequences since publication pressure may be so high that authors may be tempted to fall for this trap.

The following are some possible solutions to reduce self-plagiarism or redundant publications:

(1) Authors should be aware that plagiarism does not simply represent a scientific misconduct, but it can also have legal implications, potentially leading to criminal charges. It is not just an ethical issue; it is a violation of intellectual property rights and can be treated as a form of theft.
(2) Authors should be aware that self-plagiarism, such as redundant publications, are true scientific misconducts which should be avoided at all costs, and that an accusation of redundant publication may have extremely serious consequences for their career progression.
(3) Authors should be aware that most journals, including open access journals, have plagiarism detection tools which can be used to scan for plagiarism and self-plagiarism.
(4) The journal instructions to authors should clearly state the journal’s policy on redundant publications and plagiarism.
(5) Authors should be aware that published text, notes, and figures have a copyright associated with them which must be addressed.
(6) Pressure to publish can be overwhelming. However, authors should be aware that the number of published papers is not related to the value of a scientist. The true scientific impact of one single study may be essential to increase an author’s reputation, as one single episode of scientific misconduct will be noticed, remembered, and may nullify all their previous efforts.
